# Uniting continental thyroid societies to address challenges in thyroid health in Africa: a call to action

**DOI:** 10.1530/ETJ-25-0374

**Published:** 2025-12-09

**Authors:** Luca Persani, Ana Luiza Maia, Kirtida S Acharya, Olufemi Adetola Fasanmade, Hinde Iraqi, Chiara Centonze, Peter A Kopp, Ashok Bhaseen, Teofilo San Luis, Dong Yeob Shin, Jennifer A Sipos

**Affiliations:** ^1^ Department of Medical Biotechnology and Translational Medicine, University of Milan, Milan, Italy; ^2^ Department of Endocrine and Metabolic Diseases, IRCCS Istituto Auxologico Italiano, Milan, Italy; ^3^ Thyroid Unit, Hospital de Clinicas de Porto Alegre, Universidade Federal do Rio Grande do Sul, Porto Alegre, Brazil; ^4^ MP Shah Hospital, Nairobi, Kenia; ^5^ Department of Medicine, Faculty of Clinical Sciences, College of Medicine, University of Lagos, Yaba, Nigeria; ^6^ Department of Endocrinology, Diabetology and Nutrition, CHU Ibn Sina, Faculty of Medicine and Pharmacy, Mohammed V University, Rabat, Morocco; ^7^ CM&E EMEA Medical Director, Merck Serono, Rome, Italy; ^8^ Division of Endocrinology, Diabetes and Metabolism, University Hospital of Lausanne and University of Lausanne, Lausanne, Switzerland; ^9^ Thyroid Federation International, Bath, Canada; ^10^ Iodine Global Network in the Philippines, Metro Manila, Philippines; ^11^ Division of Endocrinology and Metabolism, Department of Internal Medicine, Severance Hospital, College of Medicine, Yonsei University, Seoul, Republic of Korea; ^12^ Division of Endocrinology & Metabolism, Arthur G James Comprehensive Cancer Center, Ohio State University, Columbus, USA

**Keywords:** health equity, iodine, screening programs, Africa

The International Round Table – Global Challenges in Thyroid Health took place at the International Thyroid Congress 2025 in Rio de Janeiro last June. It brought together representatives from four major international thyroid societies – the Asia and Oceania Thyroid Association (AOTA), the American Thyroid Association (ATA), the European Thyroid Association (ETA), and the Latin American Thyroid Society (LATS) – alongside endocrinology leaders from Kenya (Dr Kirtida S Acharya), Morocco (Dr Hinde Iraqi), and Nigeria (Dr Fasanmade Olufemi Adetola).

The session was co-chaired by Dr Luca Persani (ETA) and Dr Ana Luiza Maia (LATS), with participation from Dr Dong Yeob Shin (AOTA), Dr Jennifer Sipos (ATA), and Dr Ashok Bhaseen, President of the International Thyroid Federation (ITF) (Fig. 1).

**Figure 1:**
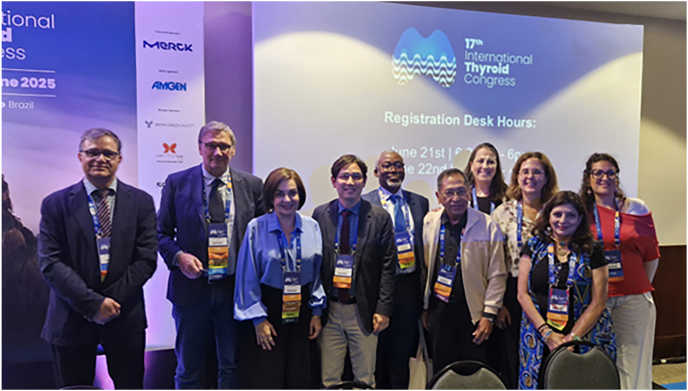
Participants of the round table from left to right: A Bhaseen, L Persani, AL Maia, DY Shin, OA Fasanmade, T San Luis Jr, J Sipos, H Iraqi, KS Acharya, and C Centonze.

The goal was to gain a better understanding of the unmet needs in diagnosing and managing thyroid disorders in African countries and to explore how the four sister societies could support efforts to overcome these barriers.

## Nigeria: data gaps and underdiagnosis

Dr Fasanmade Olufemi Adetola (Nigeria) presented the scenario in his country, noting that the biggest challenge is access to medications, tests, and accurate information. He thanked the ITC for the support in enabling their participation in Brazil and said that sharing these difficulties with a global audience is a key first step. He explained that there is a significant lack of thyroid-related data in comparison with other diseases, such as diabetes.

*‘*Few studies show the endemic nature of goiters in Nigeria. Hypothyroidism and hyperthyroidism are underdiagnosed. Government initiatives focus only on the iodization of salt*’.* He emphasized the need for more epidemiological studies and awareness, stressing that *‘*giving a voice to thyroid patients is essential – just as it is for those living with diabetes*’.*

## Morocco: unequal access and the need for screening

Dr Hinde Iraqi, who collaborates with Morocco’s Ministry of Health on programs related to diabetes and hypothyroidism, shared several core concerns in her country, such as unequal access to diagnosis, iodine deficiency, lack of systematic neonatal screening, and the absence of organized patient advocacy.

She reported that *‘*limited availability of basic thyroid tests (TSH, FT4) outside the major urban areas*’* makes early diagnosis difficult. Ultrasound is available in most cities, and scintigraphy in the major urban centers.

The high cost of thyroid testing and delays in diagnosis are major problems, and although salt iodization has been introduced, coverage remains incomplete and the mountainous geography hinders access to health services.

*‘*This remains a critical public health gap that deserves urgent action*’,* she said. Dr Hinde also noted the absence of structured patient representation: *‘*There is no structured thyroid patient association at the national level and no representation of thyroid patients in health policy or awareness campaigns*’.* To address this, informal Facebook communities are used to raise awareness.

## Kenya: ‘in every challenge lives a greater opportunity’

Dr Kirtida S Acharya brought a message of hope and resilience, opening her presentation by singing the famous song *‘*Hakuna Matata*’,* whose message is about overcoming adversity and focusing on the present. She stated, *‘*It is Africa’s time*’* and proposed several actions to change the current landscape: consolidating research, training healthcare professionals and patients through online and outreach efforts, implementing salt iodization programs across the continent, and distribution of educational materials.

She also stressed the need for lower-cost diagnostics, validated laboratory reports, telemedicine expansion, and empowering patients through knowledge. Additional proposals included widespread neonatal screening for thyroid dysfunction, the development of local clinical guidelines, and national partnerships with ministries of health and non-governmental organizations (NGOs) to better manage thyroid disorders.

### Common challenges across the continent

The need for education, limited access to healthcare providers and therapies, excessive costs for biochemical tests and equipment leading to missed or extremely delayed diagnoses, and inadequate treatments were recurring themes throughout the presentations. Isolated initiatives of key African physicians, including the participants of this round table, aiming to influence politicians or governmental actions have repeatedly fallen into the void. Thus, all participants agreed that creating a dedicated scientific thyroid society for African countries in collaboration with thyroid patient associations could be a key step toward addressing these systemic issues and obtaining support from local administrations.

Dr Luca Persani commented that *‘*the difficulties described are similar across most, if not all, African countries*’* and added, *‘*we know these issues are shared by several countries outside Africa as well*’.* Health equity and accessibility need special attention, especially on prevalent and impactful diseases, such as thyroid disorders, which are estimated to affect 200 million people worldwide. These concerns have also been summarized in an editorial published more than 10 years ago (1).

Dr Persani opened the floor for discussion, encouraging the audience to contribute ideas and share their own experiences. Among the proposed solutions were the development of educational programs by international societies and the creation of tailored clinical guidelines to improve healthcare delivery in resource-limited settings.

## Moving toward solutions

Dr Ashok Bhaseen emphasized the strength of collective action, stating that *‘*this is a great opportunity to discuss the problem and identify opportunities for solutions*’.* Dr Jennifer Sipos echoed the importance of collaboration, advocating for the formation of an African thyroid society: *‘*We need to work together to help Africa, starting by understanding the core of the problem*’.*

According to Dr Ana Luiza Maia, the session concluded with *‘*practical and potentially transformative outcomes*’.* She emphasized that it provided key insights into the specific needs of African regions and marked an essential step toward establishing a dedicated thyroid society for the continent.

Dr Dong Yeob Shin, reflecting on his visits to medical schools, stressed that *‘*education is a fundamental tool in this process*’.*

## A call to action: first steps toward a larger movement

Following the presentations, the session opened to the audience, inviting additional contributions to begin shaping a collaborative roadmap for improving thyroid care across Africa. The round table left participants energized, with a sense of shared purpose and a clear message: the time to act is now!

The following priorities have been proposed:

– Implementation of salt iodization programs following the example of Kenya. According to available sources, Kenya mandates and has achieved a high rate of iodized salt consumption, with over 99% of households using adequately iodized salt. Legislation for mandatory salt iodization was passed in 1978 and revised in 1988, and the Foods, Drugs and Chemical Substances Act requires table salt to contain a minimum amount of potassium iodate to prevent iodine deficiency disorders (2).
– The generation of education materials in multiple languages (including English, French, Swahili, Arabic, and Portuguese) to reach caregivers across diverse African subregions. These materials should be adequate for most of the African settings and comprehensive wherever possible:
  ￮ Educational events with live sessions to collect comments and questions.
  ￮ Recorded materials, with free access on a dedicated platform.
– The generation of an African section of the International Thyroid Federation of patient associations.
– The preparation of an official document illustrating the known epidemiology of thyroid disorders and the consequences of missed diagnoses, as well as proposed means to prevent and reduce their impact on the population. This document could constitute the basis for the creation of an African Thyroid Association.
– Developing clinical guidelines tailored to African countries (and other resource-limited countries). These guidelines should focus on diagnosis and management of the major thyroid disorders and address key steps for diagnosis and treatment across all age groups. Dr Kopp informed the panel of an existing initiative for the implementation of newborn screening in Algeria; notably, at this stage, none of the countries on the African continent has a functional national screening program. In this context, the need for affordable point-of-care testing (TSH) has also been emphasized.
– Implementation of actions such as the ‘Thyro-Mobile’ program to reach rural areas within the African continent, bringing healthcare professionals to do clinical assessment, point-of-care tests and portable ultrasound imaging, and doing quantitative iodine testing of household salt will increase thyroid health awareness and enhance healthcare delivery to vulnerable communities.
– Inclusion of thyroid diseases among the non-communicable diseases recognized by the World Health Organization, defined as illnesses that are not transmitted from person to person, such as most cardiovascular diseases and diabetes (3). Non-communicable diseases account for the majority of deaths globally and are closely linked to lifestyle risk factors, including unhealthy diets, and genetic predispositions that also play a key role in the development of thyroid diseases. Recognizing this connection would strongly help address thyroid diseases at the global level and in Africa specifically.

This group of experts believes that these actions should be supported by specific programs from the four thyroid sister associations AOTA, ATA, ETA, and LATS, together with a specific effort by the ITF and other organizations with a global outreach, such as the World Congress of Thyroid Cancer (WCTC).

## Declaration of interest

The authors declare that there is no conflict of interest that could be perceived as prejudicing the impartiality of the work reported.

## Funding

This work did not receive any specific grant from any funding agency in the public, commercial, or not-for-profit sector.

## References

[1] Editorial. The untapped potential of the thyroid axis. Lancet Diabetes Endo 2013 1 163. (10.1016/S2213-8587(13)70166-9) 24622353

[2] Githinji GG, Njine N, Njihia J, et al. Potassium iodate levels in processed edible salts available in retail shops throughout Kenya, 2013. Public Health Nutr 2018 21 2482–2484. (10.1017/S1368980018000915) 29669614 PMC10260749

[3] WHO 2025 Noncommunicable diseases. (https://www.who.int/news-room/fact-sheets/detail/noncommunicable-diseases)

